# Supplementary material for: β2-Integrin Adhesive Bond Tension under Shear Stress Modulates Cytosolic Calcium Flux and Neutrophil Inflammatory Response
Source: Cells. 2022 Sep 9;11(18):2822. doi: 10.3390/cells11182822 (PMC9497066; doi:10.3390/cells11182822)
Supplement: Supplementary file 1 [file cells-11-02822-s001.zip › cells-1856745-supplementary.pdf]

β2-integrin adhesive bond tension under shear stress modulates cytosolic calcium flux and neutrophil inflammatory response

Vasilios Aris Morikis [1], Szu Jung Chen [1], Julianna Madigan [1], Myung Hyun Jo [2], Lisette Caroline Werba [1],
Taekjip Ha [2,3] and Scott I. Simon [1,*]

[1] Department of Biomedical Engineering, University of California-Davis, Davis, CA 95616, USA
[2] Department of Biophysics & Biophysical Chemistry, Baltimore, MD 21218, USA
[3] Biomedical Engineering, Johns Hopkins University, Baltimore, MD 21218, USA
* Correspondence: sisimon@ucdavis.edu

Supplementary Material

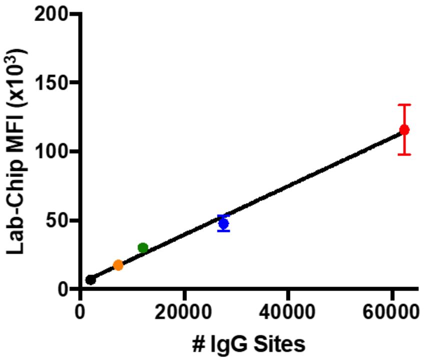

Figure S1. Determination of antibody binding sites using calibration beads with indicated fluorescence for each bead with defined number of IgG sites, settled on a glass coverslip within vascular mimetic microfluidic channels using wide-field fluorescence microscopy. MFI of each bead bound with AF488 mAb24 HA LFA-1 reporter was calculated and plotted versus the receptor number binding capacity. (

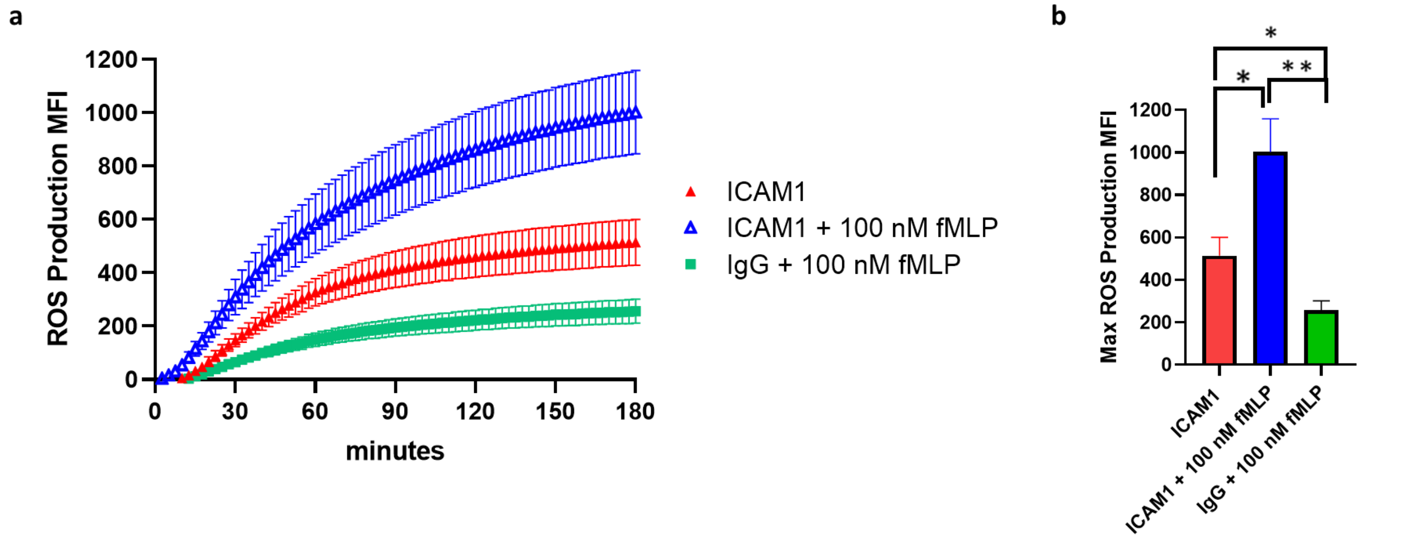

Figure S2. (a) Real time kinetics of ROS production in DHR buffer for neutrophils bound to substrate coated with ICAM-1 or IgG1 isotype control antibody, stimulated with 100 nM fMLP. Cells were shear mixed every 2.5 mins. **(b)** Quantification of maximum ROS production (n=3, * significant difference compared with ICAM-1 $p < 0.05$, ** represents significance compared with IgG isotype control $p < 0.01$)

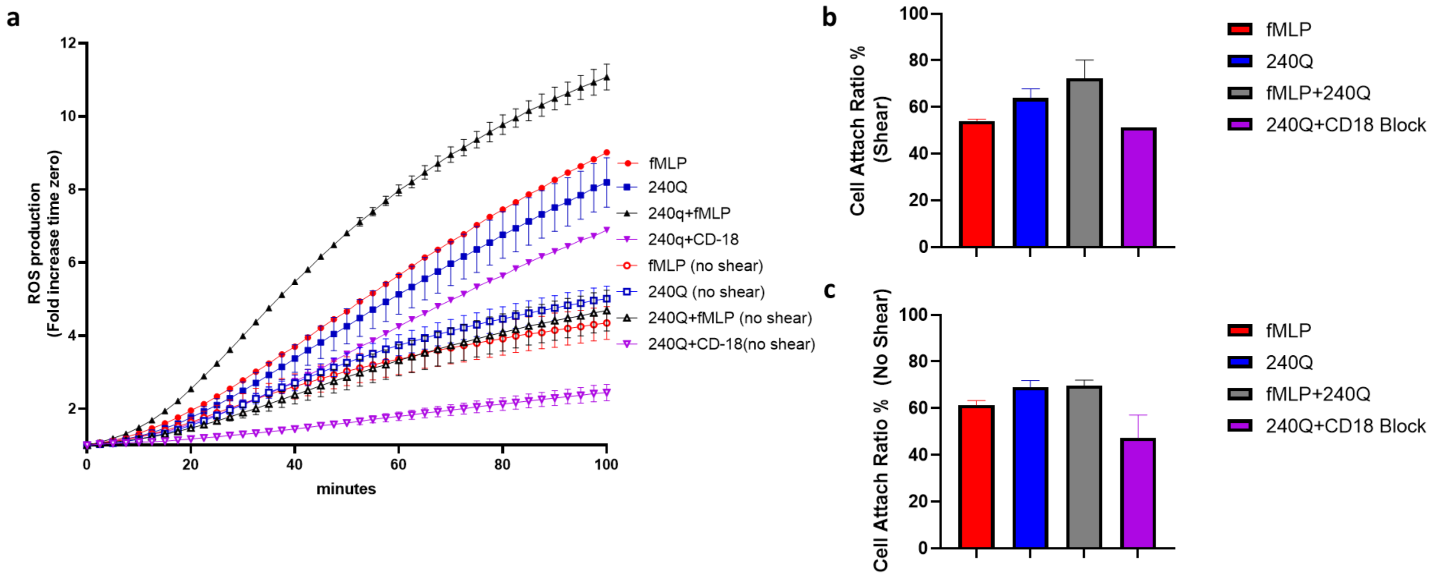

Figure S3. (a) Real time kinetics of ROS production in DHR buffer for neutrophils exposed to fMLP and/or HA-LFA-1 inducing antibody and/or CD18 blocking antibody. Cells were shear mixed every 2.5 mins or with no mixing at all. Neutrophil attachment ratio (# PMN remaining adherent at 100 min / #PMN added to each well): **(b)** sheared or **(c)** without shear measured as fraction of total input remaining at the end of the time course based upon Hoechst fluorescence.

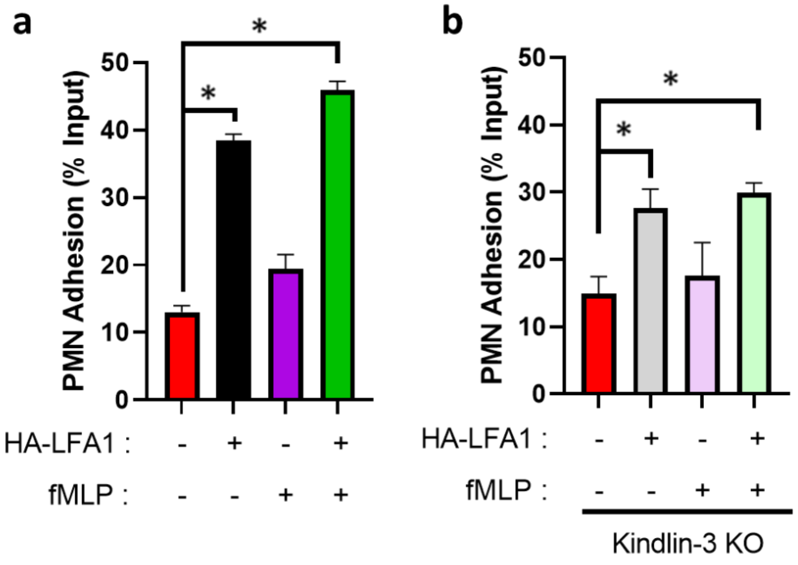

Figure S4. Neutrophils bound to ICAM-1 in the presence of HA inducing β_2-integrin antibody and/or fMLP. Cells were shear mixed every 2.5 mins or with no mixing at all. Neutrophil attachment ratio (# PMN remaining adherent at 100 min / #PMN added to each well): **(a)** CTRL R566A Kindlin-3 mutations and **(b)** Kindlin-3 siRNA KO measured as fraction of total input remaining at the end of the time course based upon Hoechst fluorescence (*, $p < 0.05$, n=4). All data are represented as mean +/- SEM

Video S1. Representative videos showing neutrophils adhered to 54pN (top), 33pN (middle), and 12pN (bottom) experiencing shear. Intracellular Ca^{2+} was measured using intracellular ratiometric calcium reporter Fura-2 (red = high Ca^{2+}, blue = low Ca^{2+}).

Video S2. Representative field of view showing neutrophils adhered to 54pN (top), 33pN (middle), and 12pN (bottom) experiencing shear. Intracellular Ca^{2+} was measured using relative intracellular calcium reporter Rhod-2 (yellow).
